# Multipoint TvDAAO Mutants for Cephalosporin *C* Bioconversion

**DOI:** 10.3390/ijms20184412

**Published:** 2019-09-07

**Authors:** Denis L. Atroshenko, Mikhail D. Shelomov, Sophia A. Zarubina, Nikita Y. Negru, Igor V. Golubev, Svyatoslav S. Savin, Vladimir I. Tishkov

**Affiliations:** 1 Department of Chemistry, Lomonosov Moscow State University, 119991 Moscow, Russia (D.L.A.) (M.D.S.) (S.A.Z.) (N.Y.N.) (I.V.G.) (S.S.S.); 2 Innovations and High Technologies MSU Ltd., 109559 Moscow, Russia; 3 Bach Institute of Biochemistry, Federal Research Centre “Fundamentals of Biotechnology” of the Russian Academy of Sciences, 119071 Moscow, Russia

**Keywords:** d-amino acid oxidase, *Trigonopsis variabilis*, cephalosporin *C*, multipoint mutants, oxidative stability, thermal stability

## Abstract

d-amino acid oxidase (DAAO, EC 1.4.3.3) is used in many biotechnological processes. The main industrial application of DAAO is biocatalytic production of 7-aminocephalosporanic acid from cephalosporin *C* with a two enzymes system. DAAO from the yeast *Trigonopsis variabilis* (TvDAAO) shows the best catalytic parameters with cephalosporin *C* among all known DAAOs. We prepared and characterized multipoint TvDAAO mutants to improve their activity towards cephalosporin *C* and increase stability. All TvDAAO mutants showed better properties in comparison with the wild-type enzyme. The best mutant was TvDAAO with amino acid changes E32R/F33D/F54S/C108F/M156L/C298N. Compared to wild-type TvDAAO, the mutant enzyme exhibits a 4 times higher catalytic constant for cephalosporin *C* oxidation and 8- and 20-fold better stability against hydrogen peroxide inactivation and thermal denaturation, respectively. This makes this mutant promising for use in biotechnology. The paper also presents the comparison of TvDAAO catalytic properties with cephalosporin *C* reported by others.

## 1. Introduction

d-amino acid oxidase (DAAO, EC 1.4.3.3) is a FAD-containing enzyme that catalyzes oxidation of d-amino acids to the corresponding α-ketoacids coupled to the production of hydrogen peroxide and ammonia [1,2,3]. DAAO is an industrially important enzyme, which is used in different biotechnological processes [4]. The major one is the production of 7-aminocephalosporanic acid (7-ACA) from cephalosporin *C* (CPC). The annual 7-ACA production is more than 2000 tons and total market volume is more than 400 million USD. 7-ACA is a precursor of many semi-synthetic cephalosporin antibiotics of different generations, including cephotaxim, ceftriaxon, cephazoline, cephoperaxon and cephixim. The total market of cephalosporin antibiotics is estimated at 10 billion USD [5,6].

Nowadays there are several processes to produce 7-ACA. The oldest one is the chemical method developed in 1970s; however, the method does not satisfy ecological requirements [6]. That is the reason to develop a biocatalytic method based on a bi-enzyme system including DAAO and glutaryl-7-ACA acylase (GAC). DAAO catalyzes CPC oxidation to α-ketoadipil-7-aminocephalosporanic acid (KA-7-ACA). Then, KA-7-ACA is spontaneously decarboxylated to glutaryl-7-aminocephalosporanic acid (GL-7-ACA) by hydrogen peroxide, the other product of DAAO reaction. Then, the second enzyme, GAC, hydrolyses GL-7-ACA to 7-ACA. Compared to the chemical method, this process provides a decrease in waste up to 10-fold and a 2-fold lower production cost [6]. Unfortunately, the process possesses several drawbacks: a complicated system with a two-pot reaction chamber due to different operational conditions for the enzymes, a relatively low GAC activity with GL-7-ACA and a low operational stability of DAAO due to oxidation by hydrogen peroxide [7]. To address the problem with hydrogen peroxide, a three-enzymatic system DAAO–GAC–catalase has been developed [8]. Recently, a new single-pot method has been proposed with the use of cephalosporin *C* acylase instead of glutaryl-7-ACA acylase [9,10,11,12]. Cephalosporin acylase is a recently-discovered enzyme capable of catalyzing direct conversion of CPC and GL-7-ACA to 7-ACA. However, its catalytic parameters with CPC are much worse in comparison with those for DAAO. Thus, there is still a need for an improved DAAO for the process of GL-7-ACA production. Therefore, an increase in DAAO catalytic properties and its operational stability is still an important industrial task.

d-amino acid oxidase from the yeast *Trigonopsis variabilis* (TvDAAO) is the most stable and active enzyme towards CPC among all known DAAOs [13]. To improve catalytic properties with CPC, mutant TvDAAOs with amino acid changes Phe54Ser, Phe54Tyr and Phe54Ala were prepared and studied [14,15]. All above substitutions showed increased catalytic properties with CPC and d-amino acids with bulky side-chains.

Hydrogen peroxide produced in the reaction of d-amino acids oxidation catalyzed by TvDAAO is the major reason for the enzyme inactivation in the reaction course. In the case of CPC oxidation, the enzymatically produced H_2_O_2_ is mainly consumed via non-enzymatic decarboxylation of KA-7-ACA followed by the enzyme reaction. A portion of the enzymatically produced hydrogen peroxide is also nonspecifically decomposed. That is why an excess of exogenous H_2_O_2_ is added to the reaction mixture to get a 100% conversion of KA-7-ACA to GL-7-ACA. This hydrogen peroxide can lead to oxidation and inactivation of TvDAAO. The highest stabilization of TvDAOO under anaerobic storage conditions is achieved by addition of SH-containing reagents (β-ME, DTT and GSH) in the presence of EDTA [16]. This fact points to TvDAAO inactivation predominantly via oxidation of cysteine residues. Thus, an increase in the enzyme stability against hydrogen peroxide is also a solution for the important task of improving the enzyme operational and storage stability.

The TvDAAO titration with Ellman’s reagent led to TvDAAO inactivation accompanied with the modification of 2 cysteine residues per TvDAAO subunit. Modification of cysteine residues by monobromobiman showed that Cys298 was the most reactive cysteine residue [16]. The loss of TvDAAO activity during the oxidation with Fe^2+^ and H_2_O_2_ occurred mainly due to the oxidation of Cys298 [17]. Slavica et al. demonstrated that oxidation of C108 residue led to a four-fold decrease in TvDAAO activity and a two-fold decrease in thermal stability [18]. Site-directed mutagenesis of Cys108 residue (amino acid changes Cys108Ser and Cys108Asp) led to decreased TvDAAO activity and stability [19]. In our laboratory, we prepared TvDAAO mutants with Cys108Ala, Cys108Ser and Cys108Phe substitutions [20,21,22]. Mutant TvDAAO Cys108Ala and TvDAAO Cys108Phe showed a 3‒4-fold increased catalytic efficiency with CPC compared to the wild-type enzyme. The amino acid change Cys108Phe also provided an increase in thermal stability.

There were attempts to increase the oxidative stability of TvDAAO by substitution of methionine residues as reported in [23]. According to Ju et al. [23], mutant TvDAAOs with substitutions Met104Leu, Met156Leu and Met209Leu showed an increased stability towards hydrogen peroxide. However, our results for TvDAAO with the same substitutions were different [24,25]. It was found that substitutions Met104Leu, Met156Leu and Met209Leu did not change TvDAAO stability towards inactivation with hydrogen peroxide. Mutation Met156Leu resulted in a 2‒3-fold increase in thermal stability whereas the substitutions Met104Leu and Met209Leu did not change this parameter.

We also carried out rational design experiments to optimize the structure of the TvDAAO FAD-binding domain: a double E32R/F33D substitution led to a 4-fold increase in the enzyme thermal stability compared to the wild-type TvDAAO [26].

In this study, we combined various positive amino acid changes into multipoint mutants. The resulting new TvDAAO mutants showed increased stability and improved activity with CPC making those newly designed mutant enzymes very promising as new biocatalysts for 7-ACA production. We combined and compared our data on TvDAAO catalytic properties with CPC with those reported by the other authors. Results obtained in our work opened the possibility of further development of new TvDAAOs with improved properties.

## 2. Results

### 2.1. Comparison of Two Methods for Determination of TvDAAO Concentration

**Figure 1:**
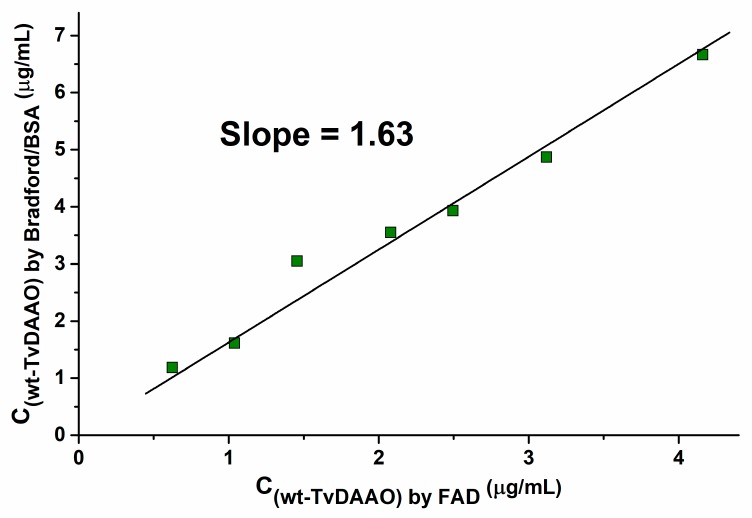
Correlation between wt-TvDAAO concentrations measured with the Bradford method with bovine serum albumin (BSA) as a standard and FAD extinction coefficient (0.1 M potassium phosphate buffer pH 8.0).

The reported catalytic parameters of TvDAAO in reaction with CPC vary considerably among different research groups. One of the reasons for this fact is the use of different methods for the determination of TvDAAO concentration. The two common procedures are (1) the Bradford method with bovine serum albumin (BSA) as a standard and (2) the use of cofactor extinction coefficient for the FAD-bound enzyme [13]. To clarify the reason for this contradiction we first checked the ratio of wt-TvDAAO concentrations measured using the Bradford/BSA method and FAD extinction coefficient (Figure 1). As can be seen, there is a linear correlation between the concentrations determined by the two methods, with the ratio C_(wt-TvDAAO) by FAD_/C_(wt-TvDAAO) by Bradford/BSA_ of ca. 1.63, which perfectly explains the discrepancy on the enzyme parameters reported in the literature.

### 2.2. Preparation of Miltipoint TvDAAO Mutants

Three types of mutations were used to prepare multipoint TvDAAO mutants with improved properties. Glu32Arg/Phe33Asp and (or) Met156Leu substitutions would provide an enzyme with an increased thermal stability, Phe54Ser, with the improved catalytic parameters in CPC oxidation reaction, and Cys108Phe and (or) substitutions of cysteine in 298 position, with the increased thermal and oxidative stability. Multipoint mutants of TvDAAO with the following substitutions were obtained:

- E32R/F33D/C108F (abbreviation used in the manuscript RDF)
- E32R/F33D/F54S/C108F (RDSF)
- F54S/C108F/M156L (SFL)
- E32R/F33D/F54S/C108F/M156L (RDSFL)
- E32R/F33D/F54S/C108F/M156L/C298G (RDSFLG)
- E32R/F33D/F54S/C108F/M156L/C298N (RDSFLN)
- E32R/F33D/F54S/C108F/M156L/C298Q (RDSFLQ)

Mutants were expressed in *E. coli* cells and purified using standard chromatography techniques [27]. All mutants were expressed in the active soluble form. The results of purification are shown in Table 1. The yield was calculated as the ratio of the total activity of a purified enzyme to the total activity in a crude extract. The purity of TvDAAO mutants was more than 90% based on SDS-PAGE.

### 2.3. Thermal Stability of TvDAAO Mutants

**Figure 2:**
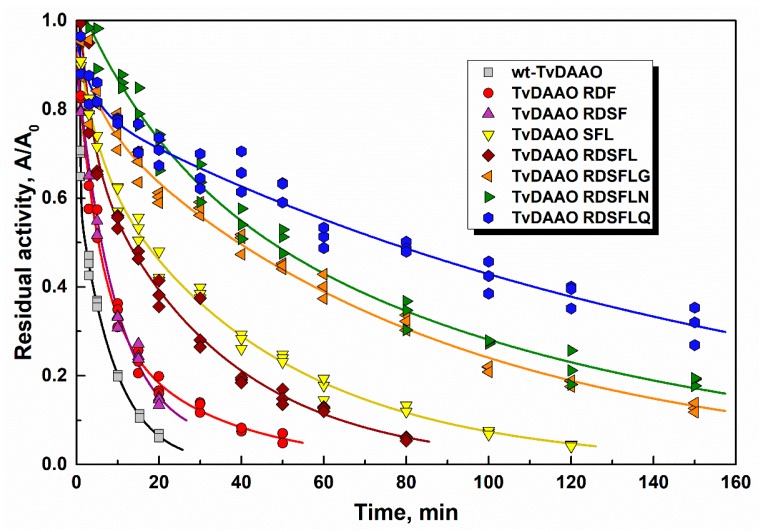
Inactivation time-course for the wild-type TvDAAO and mutant enzymes at 60 °C (0.1 M potassium phosphate buffer pH 8.0). Enzyme concentration is 10 μg/mL.

Thermal stability of TvDAAO mutants was studied through inactivation kinetics in 0.1 M potassium phosphate buffer (KPB), pH 8.0, at different temperatures. The initial concentration of all enzymes was equal to 10 µg/mL because TvDAAO inactivation is concentration dependent [20,28]. The time-course of residual activity of new mutant TvDAAOs at 60 °C is shown in Figure 2. All TvDAAO mutants are more stable than the wild-type enzyme. The thermal stabilities of TvDAAO RDF and TvDAAO RDSF were approximately the same. Three of the most stable mutants (TvDAAO RDSFLG, TvDAAO RDSFLN and TvDAAO RDSFLQ) had cysteine substitution in 298 position.

Half-life times τ**_1/2_** for TvDAAO mutants and wt-TvDAAO are presented in Table 2. Mutant TvDAAOs with RDF and RDSF substitutions on average are 3–4 times more stable and TvDAAO SFL and TvDAAO RDSFL ~10-fold more stable than wt-TvDAAO. TvDAAO mutants with cysteine substitution in the 298 position show a 15–30 times higher thermal stability. TvDAAO RDSFLQ showed a rapid 25% decrease in activity in the beginning, but later the inactivation rate became significantly slower. At 66 °C TvDAAO RDSFLQ loses half of its activity within the first minute, but then remains active up to 30 min. The various inactivation kinetics of mutant TvDAAOs is likely due to different mechanisms of enzyme stabilization resulting from the amino acid changes. A number of various inactivation mechanisms for TvDAAO were proposed before such as oligomer dissociation, FAD dissociation from the active site and irreversible thermal denaturation [2,14,20,28].

### 2.4. Stability of Mutant TvDAAOs Against Hydrogen Peroxide

**Figure 3:**
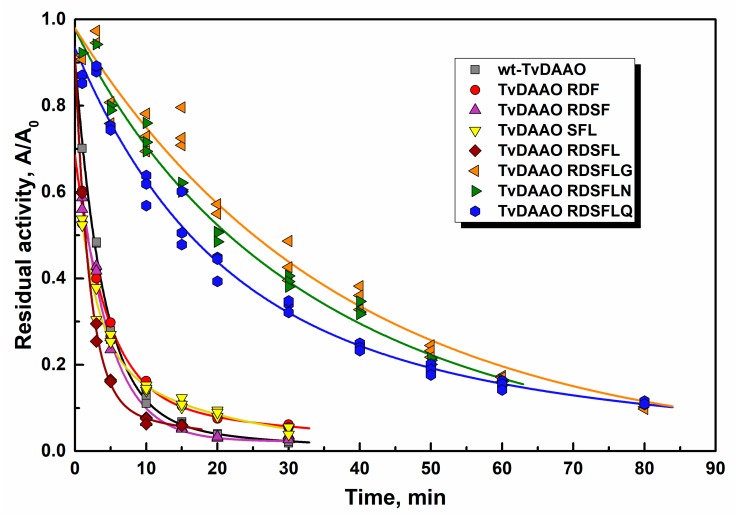
Inactivation time-course for TvDAAO mutants and wild-type enzyme in 0.1 M H_2_O_2_ (0.1 M potassium phosphate buffer pH 8.0, 30 °C). Enzyme concentration is 10 μg/mL.

Low stability against oxidation by hydrogen peroxide is one of the main restrictions for TvDAAO application. Figure 3 shows inactivation of the enzymes in the presence of 0.1 M hydrogen peroxide in KPB, pH 8.0. The half-life times for mutant TvDAAOs and wild-type enzyme in 0.1 M and 0.01 M hydrogen peroxide are presented in Table 3. All mutant enzymes with Cys108Phe substitution have stability comparable with that for the wild-type TvDAAO. Moreover, inactivation kinetics of the wild-type enzyme by H_2_O_2_ does not follow the pseudo-first order kinetics. These data indicate the presence of at least two residues essential for oxidative stability. Amino acid changes only in the 298 position increased stability against hydrogen peroxide inactivation by 1.5–2.5 times only (to be published). Substitutions of cysteine residue in the 108 and 298 positions simultaneously (TvDAAO RDSFLG, TvDAAO RDSFLN and TvDAAO RDSFLQ) resulted in a 6–10-fold increased stability in the presence of hydrogen peroxide. This means that oxidation of cysteine residue in the 298 position is crucial for wt-TvDAAO oxidative inactivation, whereas Cys108 oxidation determines the inactivation rate only for TvDAAOs with Cys298 substitutions. The inactivation kinetics for TvDAAO RDSFLG, TvDAAO RDSFLN and TvDAAO RDSFLQ can be described by pseudo-first order kinetics, which indicates a single process leading to the inactivation of these enzymes.

### 2.5. Kinetic Properties of Mutant TvDAAOs with d-Amino Acids

**Figure 4:**
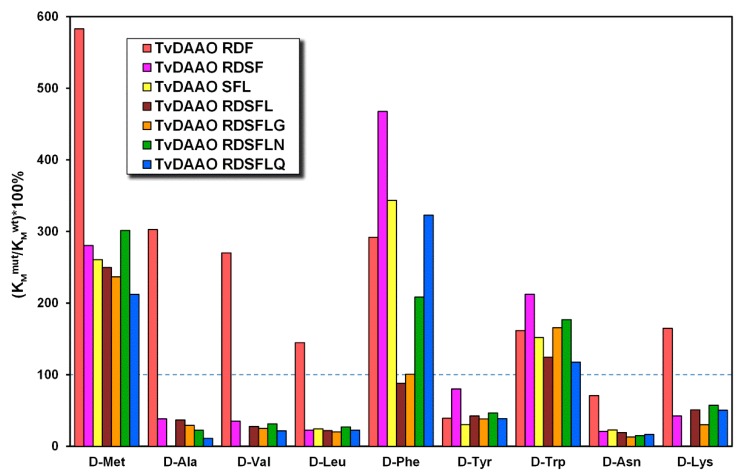
Relative change in Michaelis constants ((*K_M_*^mut^/*K_M_*^wt^)*100%) of TvDAAO mutants. The Michaelis constant of the wild-type enzyme was taken as 100% and indicated by the blue dashed line (0.05 M potassium phosphate buffer pH 8.0, 30 °C, air saturated).

**Figure 5:**
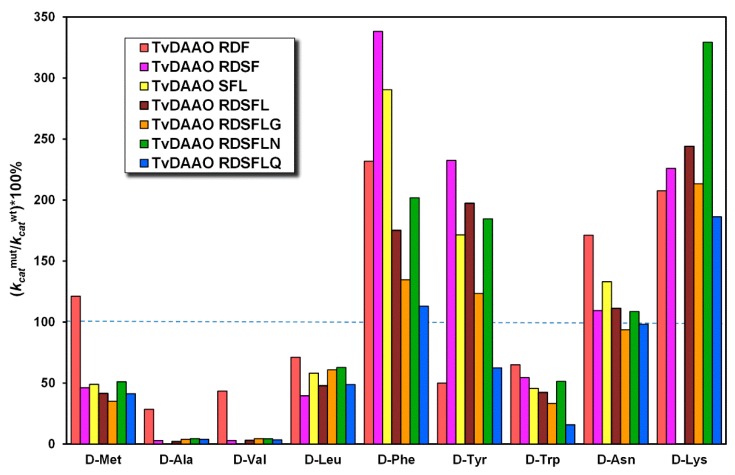
Relative change in catalytic constants ((*k_cat_*^mut^/*k_cat_*^wt^)*100%) of TvDAAO mutants. The catalytic constant of the wild-type enzyme was taken as 100% and indicated by the blue dashed line (0.05 M potassium phosphate buffer pH 8.0, 30 °C, air saturated).

Kinetic parameters of mutant TvDAAOs with different d-amino acids are presented in Table A1 and Table A2. For better perception we presented a relative change in Michaelis constant ((*K_M_*^mut^/*K_M_*^wt^)*100%) and catalytic constant ((*k_cat_*^mut^/*k_cat_*^wt^)*100%) for mutant TvDAAOs (Figure 4 and Figure 5, respectively). The Michaelis constant and catalytic constant of the wild-type TvDAAO were taken as 100% and are indicated by the blue dashed lines. The substrate specificity profiles were similar for TvDAAOs containing Phe54Ser substitution (for all except TvDAAO RDF); Michaelis constants decreased for most d-amino acids (d-Ala, d-Val, d-Leu, d-Tyr, d-Asn and d-Lys). A common observation was an increase in catalytic constants for d-Phe, d-Tyr, d-Asn and d-Lys and a decrease for d-Met, d-Ala, d-Val, d-Leu and d-Trp.

### 2.6. Kinetic Properties of Mutant TvDAAOs with Cephalosporin C

Catalytic parameters with CPC were determined with High Performance Liquid Chromatography (HPLC) using the initial velocities approach. Results are presented in Table 4. Catalytic constants increased by 3–5 times for TvDAAO mutants with Phe54Ser substitution, whereas the Michaelis constants remained practically the same as that of the wild-type enzyme. Mutants TvDAAO SFL, TvDAAO RDSFL and TvDAAO RDSFLN had the highest values for the catalytic constant (119, 106 and 121 s^−1^, respectively).

## 3. Discussion

A correct determination of enzyme concentrations is very important to calculate real catalytic constants of an enzyme. Various methods for the determination of TvDAAO concentration can be found in the literature. Here, we studied the correlation between the two basic methods, the Bradford method and the extinction coefficient for active-site bound FAD. Despite the fact of the linear correlation between the two methods, the slope value (ratio C_(wt-TvDAAO) by FAD_/C_(wt-TvDAAO) by Bradford/BSA_ ) is not 1, but 1.63. This ratio can be used for the evaluative comparison of catalytic constants obtained by different authors. Actually, *k_cat_* values reported in the works based on the Bradford/BSA method for the determination of protein concentration are understated by the factor of 1.63. We compared TvDAAO catalytic parameters with CPC from different reports in Table 5 and evaluated the apparent catalytic constants using the ratio C_(wt-TvDAAO) by FAD_/C_(wt-TvDAAO) by Bradford/BSA_ = 1.63 and MW_(wt-TvDAAO)_ = 39.3 kDa (see column “Recalculated catalytic properties” in Table 5).

The recalculated values of the catalytic constants are still very different. This may be due to the different conditions of CPC oxidation and methods of reaction rate measurement. The high value *k_cat_* = 72 s^−1^ reported in [13] may reflect the use of exogenous FAD in the reaction to stabilize TvDAAO or lead to activation of apo-TvDAAO, if any was present. An assay of TvDAAO activity with CPC by hydrogen peroxide release using *o*-phenylenediamine (OPD) and horseradish peroxidase (HRP) [30] is not a reliable method because hydrogen peroxide, the product of DAAO-catalyzed reaction, is consumed in the following reaction of non-enzymatic KA-7-ACA decarboxylation.

We obtained multipoint mutants of TvDAAO with increased thermal and oxidative stability and improved catalytic properties in cephalosporin *C* oxidation. High thermal stability of enzymes can simplify purification procedures due to the possibility of heat treatment. Thermostable enzymes can be used in processes at elevated temperatures, which provide an antimicrobial environment and a higher enzyme activity. All new TvDAAO mutants showed better thermal stability than wt-TvDAAO. The highest stabilization effect (15‒30 times) was observed for mutant TvDAAOs with substitutions RDSFLG, RDSFLN and RDSFLQ.

One of the major drawbacks of wt-TvDAAO is a low stability against inactivation by hydrogen peroxide, which is the product of reactions catalyzed by DAAO. Inactivation of many enzymes by oxidants is related to oxidation of cysteine and (or) methionine residues. Long-term stability of TvDAAO has been associated with the oxidation of sulfhydryl groups [16]. Since point mutations of Cys residues in 108 and 298 positions influenced catalytic properties and stability of TvDAA [16,18], we prepared mutant enzymes with double substitutions of both Cys residues. Enzyme form with simultaneous changes of cysteine residues in the 108 and 298 positions (TvDAAO RDSFLG, TvDAAO RDSFLN and TvDAAO RDSFLQ) have a 6–10-fold increased stability against hydrogen peroxide oxidation compared to the wild-type enzyme. This effect is higher than those observed for the single point mutations (slight effect for Cys108 and only 1.5–2.5 times for Cys298). Our observations prove that both Cys residues are essential for TvDAAO oxidative stability but their input is not equal. Cys298 is more active than the other, but after its substitution, Cys108 plays the main role in oxidative stability of TvDAAO.

The data on the substrate specificity of TvDAAO mutants can be used for selection of the most suitable enzymes for a certain process of fine organic synthesis and especially for the construction of biosensors of high specificity and sensitivity. A good example is TvDAAO mutant with Phe54Ser substitution which exhibits decreased Michaelis constants for most d-amino acids and increased catalytic constants towards some of them.

A combination of positive single mutations resulted in multipoint TvDAAO mutants which were superior for the process of cephalosporin *C* oxidation. The highest catalytic constants were observed for three mutants—TvDAAO SFL, TvDAAO RDSFL and TvDAAO RDSFLN (119, 106 and 121 s^−1^, respectively). These values are 4–5 times higher than those for wt-TvDAAO. Previously, the best TvDAAO catalytic constant with CPC was reported for mutant TvDAAO Phe54Tyr (2200 ± 570 min^−1^ [15] (recalculated *k_cat_* = 60 s^−1^). In our experiments *k_cat_* values were almost doubled.

Table 6 summarizes input of each mutation to the final properties of new multipoint TvDAAO mutants.

Based on Table 6 we can conclude that the mutant TvDAAO RDSFLN is the most promising for practical application and in particular for 7-ACA production. This mutant possesses more than 4 times higher catalytic constant in the oxidation of cephalosporin *C*, an 8-fold higher stability against hydrogen peroxide oxidation and 20 times better temperature stability compared to wt-TvDAAO. The mutants described in this article can be used for further investigation and improvement of properties.

## 4. Materials and Methods

### 4.1. Preparation of TvDAAO Mutants

TvDAAO mutants were prepared by subcloning of DNA fragments from previously obtained genes with single and double mutations. New mutants were expressed in *E. coli* and purified as described previously [27]. Concentration of TvDAAO mutants was determined by using a FAD extinction coefficient 10.8 mM^−1^·cm^−1^ at 455 nm [13] in a UV-1800PC spectrophotometer (Shimadzu, Dusseldorf, Germany). The purity of the enzyme was monitored by analytical electrophoresis in a 12% polyacrylamide gel in presence of sodium dodecyl sulfate and β-mercaptoethanol.

### 4.2. Kinetic Assay

TvDAAO activity with d-amino acids was measured using the “horseradish peroxidase method” [31] with ABTS as substrates of HRP. The activity was determined by the accumulation of the ABTS oxidized product at 414 nm (ε_414_ = 36,000 M^−1^·cm^−1^) in a UV-1800PC spectrophotometer equipped with a thermostated cuvette compartment. Reactions were carried out in 0.05 M KPB pH 8.0, 30 °C, air saturated.

TvDAAO activity with CPC was assayed CPC consumption followed by HPLC. CPC concentration was monitored using the Knauer HPLC System on a Kromasil C18 column (125 mm × 4.0 μm (KhroMass, Moscow, Russia) in 0.2 M Na-acetate, pH 5.3, 10% acetonitrile, 30 °C, 0.75 mL/min. Reactions were carried out in 1.5-mL open plastic tubes with stirring (1400 rpm) in 0.1 M KPB pH 8.0, 30 °C, air saturated. Concentration of CPC varied in the range from 0.3 to 20.0 mM. Reaction rate was calculated from the initial slope of dependence of CPC concentration on time.

The kinetic parameters *V_M_* and *K_M_* were calculated from dependence of initial reaction rate on CPC concentration by the nonlinear regression method using OriginPro 9.1 software (OriginLab Corporation, Northampton, MA, United States). The catalytic constant *k_cat_* was calculated from the *V_M_* value and enzyme concentration.

### 4.3. Thermal and Oxidation Stability

The thermal and oxidative stability of TvDAAO mutants were studied as described previously [24]. Thermal stability was determined in 0.1 M KPB, pH 8.0, and enzyme concentration 10 µg/mL. Oxidation stability was studied in 0.1 M KPB pH 8.0 30 °C in the presence of 10 or 100 mM H_2_O_2_. Concentration of H_2_O_2_ in stock solution was determined by using extinction coefficient 43.6 M^−1^·cm^−1^ at 240 nm. Enzyme concentration was 10 µg/mL and total volume of inactivation mixture was 1.5 mL. At certain time intervals samples of 100 μL were taken and mixed with 5 μL of a catalase solution (10,000 U/mL final concentration 50 U in total) to decompose the hydrogen peroxide. Then, 30 μL was taken to measure residual enzyme activity. Measurements were done at least in triplicate.

## Figures and Tables

**Table 1:** Purification of wild-type and mutant TvDAAOs.

TvDAAO Form	Yield, %	Specific Activity ^1^, (U/mg)
wild-type	44	140
RDF	44	149
RDSF	43	47
SFL	61	63
RDSFL	95	50
RDSFLG	90	55
RDSFLN	72	66
RDSFLQ	82	46

^1^ Activity measured with d-Met as a substrate.

**Table 2:** Half-life times τ_1/2_ (min) of wild-type TvDAAO and TvDAAO mutants (0.1 M potassium phosphate buffer pH 8.0).

TvDAAO form	Temperature (°C)								
	50	52	54	56	58	60	62	64	66
wild-type	73	47	23	7.2	4.7	2.7	-	-	-
RDF	-	-	150	42	19	8.9	3.1	-	-
RDSF	-	-	79	33	9.5	5.6	2.8	-	-
SFL	-	-	-	110	57	17	9.8	-	-
RDSFL	-	-	-	-	43	15	4.6	1.4	-
RDSFLG	-	-	-	-	-	41	27	6.5	2.6
RDSFLN	-	-	-	-	-	54	19	6.0	4.2
RDSFLQ	-	-	-	-	-	77	16	4.0	-

**Table 3:** Half-life times τ_1/2_ (min) of TvDAAO mutants and wild-type enzyme under hydrogen peroxide inactivation (0.1 M potassium phosphate buffer pH 8.0).

TvDAAO Form	[H_2_O_2_], (M)	
	0.01 M	0.1 M
wild-type	14	2.6
RDF	-	1.6
RDSF	-	1.8
SFL	17	1.7
RDSFL	9	1.5
RDSFLG	**120**	**25**
RDSFLN	**120**	**22**
RDSFLQ	**90**	**18**

Increased half-life time of TvDAAO mutants in comparison with wild-type enzyme is marked with bold.

**Table 4:** Catalytic parameters of mutant TvDAAOs with cephalosporin *C* (0.1 M potassium phosphate buffer pH 8.0, 30 °C, air saturated).

TvDAAO Form	*K_M_*, (mM)	*k_cat_*, (s^−1^)	*k_cat_*/*K_M_*, (s^−1^·mM^−1^)
wild-type	1.4 ± 0.4	26 ± 3	18 ± 5
RDF	**1.15 ± 0.08** ^1^	25.5 ± 1.4	22 ± 2
RDSF	1.9 ± 0.4	**88 ± 10**	**45 ± 11**
SFL	1.9 ± 0.6	**119 ± 7**	**60 ± 20**
RDSFL	1.6 ± 0.3	**106 ± 9**	**66 ± 12**
RDSFLG	**0.8 ± 0.2**	**65 ± 5**	**79 ± 15**
RDSFLN	2.9 ± 0.5	**121 ± 15**	**42 ± 8**
RDSFLQ	1.4 ± 0.3	**68 ± 0.5**	**50 ± 9**

^1^ Improved catalytic parameters of mutant TvDAAOs in comparison with wild-type enzyme are marked with bold.

**Table 5:** Comparison of reported catalytic properties of wt-TvDAAO with cephalosporin *C*.

Source	wt-TvDAAO Concentration	Reaction Condition	Measurement Methods	Catalytic Properties from Source	Recalculated Catalytic Properties ^1^
This work	FAD extinction	pH 8.0, 30 °C 100 mM NaPB	Cephalosporin *C* consumption	*K_M_* = 1.4 mM *k_cat_* = 26 s^−1^	*K_M_* = 1.4 mM *k_cat_* = 26 s^−1^
[29]	Bradford	pH 8.0, 37 °C 0.21 mM O_2_	Oxygen consumption	*K_M_* = 0.83 mM Specific activity = 46 U/mg (*k_cat_* = 30.2 s^−1^)	*K_M_* = 0.83 mM *k_cat_* = 49 s^−1^
[15]	Bradford	pH 7.5, 22 °C 50 mM NaPB	GL-7-ACA production	*K_M_* = 1.6 mM *k_cat_* = 370 min^−1^ (*k_cat_* = 6.2 s^−1^)	*K_M_* = 1.6 mM *k_cat_* = 10 s^−1^
[13]	FAD extinction	pH 8.5, 25 °C 0.2 mM FAD air saturated	Oxygen consumption	*K_M_* = 2.4 mM *k_cat_* = 4300 min^−1^ (*k_cat_* = 72 s^−1^)	*K_M_* = 2.4 mM *k_cat_* = 72 s^−1^
[30]	Bradford	pH 8.0, 25 °C 100 mM KPB	OPD/HRP	*K_M_* = 9 mM *k_cat_* = 55 s^−1^	*K_M_* = 9 mM *k_cat_* = 89 s^−1^

^1^ Data recalculated with C_(wt-TvDAAO) by FAD_/C_(wt-TvDAAO) by Bradford/BSA_ = 1.63 and MW_(wt-TvDAAO)_ = 39.3 kDa.

**Table 6:** Comparison of the properties of the TvDAAO mutants with wt-TvDAAO.

TvDAAO Form	Thermal Stability	Oxidative Stability	*k_cat_* with CPC	Total
RDF	+	0	0	+
RDSF	+	0	+	++
SFL	+	0	++	+++
RDSFL	+	0	++	+++
RDSFLG	++	++	+	+++++
RDSFLN	++	++	++	++++++
RDSFLQ	++	+	+	++++

“0”—minor effect, “+”—moderate improvement, “++”—greatest improvement. For thermal stability “+” mean 3-10 times higher stability, “++” mean 15-30 times higher stability. For oxidative stability “+” mean ≈6 times higher stability, “++” mean 8-10 times higher stability. For *k_cat_* wit CPC “+” mean ≈2.5 times higher value, “++” mean 3-5 times higher value.

## Abbreviations

DAAO	d-amino acid oxidase
7-ACA	7-aminocephalosporanic acid
CPC	Cephalosporin *C*
GAC	Glutaryl-7-ACA acylase
KA-7-ACA	α-keto adipil-7-ACA
GL-7-ACA	Glutaryl-7-ACA
TvDAAO	d-amino acid oxidase from yeast *Trigonopsis variabilis*
BSA	Bovine serum albumin
wt-TvDAAO	Wild-type TvDAAO
RDF	Mutant TvDAAO with amino acid changes E32R/F33D/C108F
RDSF	Mutant TvDAAO with amino acid changes E32R/F33D/F54S/C108F
SFL	Mutant TvDAAO with amino acid changes F54S/C108F/M156L
RDSFL	Mutant TvDAAO with amino acid changes E32R/F33D/F54S/C108F/M156L
RDSFLG	Mutant TvDAAO with amino acid changes E32R/F33D/F54S/C108F/M156L/C298G
RDSFLN	Mutant TvDAAO with amino acid changes E32R/F33D/F54S/C108F/M156L/C298N
RDSFLQ	Mutant TvDAAO with amino acid changes E32R/F33D/F54S/C108F/M156L/C298Q
SDS-PAGE	Polyacrylamide gel electrophoresis in presence of sodium dodecyl sulfate
KPB	Potassium phosphate buffer
ABTS	2,2′-azino-bis(3-ethylbenzothiazoline-6-sulphonic acid
HPLC	High pressure liquid chromatography
OPD	*o*-phenylenediamine
HRP	Horseradish peroxidase

## Appendix A

**Table A1:** Kinetic parameters of wt-TvDAAO, TvDAAO RDF, TvDAAO RDSF and TvDAAO SFL with different d-amino acids (0.05 M KPB, pH 8.0, 30 °C, air saturated).

Amino Acid	wt-TvDAAO		TvDAAO RDF		TvDAAO RDSF		TvDAAO SFL	
	*K_M_*, mM	*k_cat_*, s^−1^	*K_M_*, mM	*k_cat_*, s^−1^	*K_M_*, mM	*k_cat_*, s^−1^	*K_M_*, mM	*k_cat_*, s^−1^
d-Met	0.46	81	2.7	**98** ^1^	1.3	37	1.2	39
d-Ala	17	109	51	31	**6.4**	3.1	-	-
d-Val	14	85	39	37	**5.1**	2.5	-	-
d-Leu	0.40	29	0.58	21	**0.09**	12	**0.1**	17
d-Phe	0.37	27	1.1	**63**	1.7	**92**	1.3	**79**
d-Tyr	0.45	22	**0.18**	11	0.36	**52**	**0.14**	**39**
d-Trp	0.49	42	0.79	28	1.0	23	0.74	19
d-Asn	23	62	**16** ^1^	**107**	**4.7**	68	**5.2**	**83**
d-Lys	29	3.5	48	**7.3**	**12**	**8**	-	-
d-Ser	37	20	32	5.5	-	-	-	-
d-Thr	11	1.8	- ^2^	-	-	-	-	-

^1^ Improved catalytic parameters of TvDAAO mutants in comparison with wt-TvDAAO are marked with bold. ^2^ Too low activity.

**Table A2:** Kinetic parameters of TvDAAO RDSFL, TvDAAO RDSFLG, TvDAAO RDSFLN and TvDAAO RDSFLQ with different d-amino acids (0.05 M KPB, pH 8.0, 30 °C, air saturated).

Amino Acid	TvDAAO RDSFL		TvDAAO RDSFLG		TvDAAO RDSFLN		TvDAAO RDSFLQ	
	*K_M_*, mM	*k_cat_*, s^−1^	*K_M_*, mM	*k_cat_*, s^−1^	*K_M_*, mM	*k_cat_*, s^−1^	*K_M_*, mM	*k_cat_*, s^−1^
d-Met	1.1	33	1.1	28	1.4	41	0.98	33
d-Ala	**6.1** ^1^	2.3	**4.9**	4.2	**3.7**	4.9	**1.8**	4.3
d-Val	**4.0**	2.6	**3.6**	3.8	**4.5**	3.7	**3.1**	3.0
d-Leu	**0.09**	14	**0.08**	18	**0.11**	18	**0.09**	14
d-Phe	0.33	**48**	0.37	**37**	0.77	**55**	1.2	31
d-Tyr	**0.19**	**44**	**0.17**	**28**	**0.21**	**42**	**0.17**	14
d-Trp	0.61	18	0.81	14	0.87	22	0.58	6.7
d-Asn	**4.3**	69	**2.9**	58	**3.4**	68	**3.7**	61
d-Lys	**15**	**8.6**	**8.9**	**7.6**	**17**	**12**	**14**	**6.6**
d-Ser	**9.0**	0.98	- ^2^	-	**9.5**	2.2	-	-
d-Thr	9.8	0.47	-	-	-	-	-	-

^1^ Improved catalytic parameters of TvDAAO mutants in comparison with wt-TvDAAO are marked with bold. ^2^ Too low activity.
